# Isochrony and rhythmic interaction in ape duetting

**DOI:** 10.1098/rspb.2022.2244

**Published:** 2023-01-11

**Authors:** Teresa Raimondi, Giovanni Di Panfilo, Matteo Pasquali, Martina Zarantonello, Livio Favaro, Tommaso Savini, Marco Gamba, Andrea Ravignani

**Affiliations:** ^1^ Department of Life Sciences and Systems Biology, University of Turin, Turin, Italy; ^2^ Conservation Ecology Program, King Mongkut University of Technology Thonburi, School of Bioresources and Technology, Bangkok, Thailand; ^3^ Comparative Bioacoustics Group, Max Planck Institute for Psycholinguistics, Nijmegen, The Netherlands; ^4^ Center for Music in the Brain, Department of Clinical Medicine, Aarhus University and The Royal Academy of Music Aarhus/Aalborg, Denmark

**Keywords:** song, gibbon, synchrony, rhythm, music‌

## Abstract

How did rhythm originate in humans, and other species? One cross-cultural universal, frequently found in human music, is isochrony: when note onsets repeat regularly like the ticking of a clock. Another universal consists in synchrony (e.g. when individuals coordinate their notes so that they are sung at the same time). An approach to biomusicology focuses on similarities and differences across species, trying to build phylogenies of musical traits. Here we test for the presence of, and a link between, isochrony and synchrony in a non-human animal. We focus on the songs of one of the few singing primates, the lar gibbon (*Hylobates lar*), extracting temporal features from their solo songs and duets. We show that another ape exhibits one rhythmic feature at the core of human musicality: isochrony. We show that an enhanced call rate overall boosts isochrony, suggesting that respiratory physiological constraints play a role in determining the song's rhythmic structure. However, call rate alone cannot explain the flexible isochrony we witness. Isochrony is plastic and modulated depending on the context of emission: gibbons are more isochronous when duetting than singing solo. We present evidence for rhythmic interaction: we find statistical causality between one individual's note onsets and the co-singer's onsets, and a higher than chance degree of synchrony in the duets. Finally, we find a sex-specific trade-off between individual isochrony and synchrony. Gibbon's plasticity for isochrony and rhythmic overlap may suggest a potential shared selective pressure for interactive vocal displays in singing primates. This pressure may have convergently shaped human and gibbon musicality while acting on a common neural primate substrate. Beyond humans, singing primates are promising models to understand how music and, specifically, a sense of rhythm originated in the primate phylogeny.

## 1. Introduction

Rhythm permeates human life across physiological, behavioural and social domains: heartbeats, neural oscillations, spoken language and music are all built on precise rhythmic patterns, which act as building blocks of physiological and communicative processes [1]. This is true for humans, but how widespread is endogenous rhythm production in the animal kingdom [2]? The simplest, lowest-entropy rhythmic structure is isochrony, namely a pattern where time intervals between successive onsets of a signal all have roughly equal durations [2]. Isochronous rhythm can be produced at an individual level: the heartbeat, walking and singing are some examples. Rhythm also plays a crucial role in interactive processes, as in human music and animal coordinated displays. Inter-individual interaction has an adaptive value in animal evolution [3] rhythm, as a tool, may help individuals to coordinate by predicting the interlocutor's turn and preparing and adapting their own. This can be mediated through two opposite strategies [3,4]. The first is synchrony, where the overlap between individuals' phonation is maximized; this happens, for example, in human joint singing. The second is turn-taking, where the overlap is minimized on the advantage of rapid exchange of short turns, as, for example, in speech.

The spontaneous production of isochronous vocal patterns seems relatively rare in other vertebrates; it has only been reported in two bird species [5,6], a bat [7], rock hyraxes [8] and one primate [9]. The empirical connection between isochrony and synchrony is even less clear because the two are often studied in isolation [10]. This lack of data strikingly contrasts with the hypothesis that a link between isochrony and synchrony, potentially already present in our hominoid ancestors’ loud-calls, may be the mechanism that shaped our coordinated communication [4]. In fact, every human culture shows collective, pulse-based rhythmic singing and dancing displays, in which individuals entrain to an isochronous, predictable pulse, allowing group coordination [11]. This purported role of cooperative vocal interaction in enhancing rhythmic regularity has been detected not only in collective dance and music but also in vocalizations [12]: in humans, synchrony boosts isochrony.

**Figure 1.**
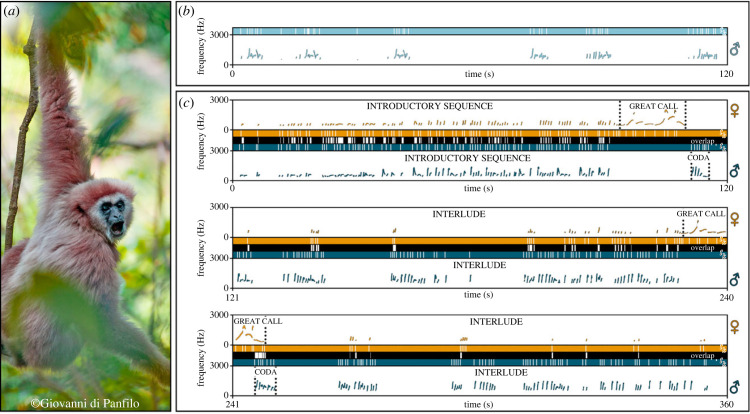
Organization of lar gibbon's songs. (*a*) Male lar gibbon singing in the Huai Kha Khaeng Wildlife Sanctuary (Thailand). (*b*) Spectrogram and inter-onset-interval graphs of the male solo. The fundamental frequency is highlighted in light blue on the spectrogram. The coloured bar indicates inter-onset intervals (*t_k_*) of the solo singing male, where solid white lines on the bar represent the onsets. (*c*) Spectrograms and inter-onset-interval graphs of the reproductive couple's whole duet. The fundamental frequency of individuals' contributions is highlighted on the spectrogram in dark blue for the male contribution to duet and dark yellow for the female contribution to duet. The sections of the song are labelled in the upper part of the spectrogram and separated with dotted lines. Coloured bars indicate inter-onset intervals (*t_k_*) of the contributions of each individual with white lines again corresponding to the onsets. Black bars turn white when the co-singers overlap. Notice how rhythmicity unfolds heterogeneously throughout the duet, alternating periods of higher and lower overlap. Note clusters onsets of the duetting gibbons influence each other (see also figure 4), with introductory sequences and interludes showing higher levels of synchrony, while great calls and codas partly overlap.

Are humans the only apes capable of flexibly orchestrating isochrony and synchrony in their behaviour? To date, the joint presence of, and connection between, isochrony and synchrony in other mammals has never been found. Here we show the first evidence for isochrony, rhythmic flexibility mediated by co-singing, and synchrony in the vocalizations of an ape, the lar gibbon (*Hylobates lar*; figure 1*a*). Gibbons are closely related to humans and share with us an unusual form of vocal communication: the song (figure 1*b,c*). Primate song plays a crucial role in various social and sexual dynamics (e.g. territorial defence, hierarchies and partnership assessment, courtship, social bonding, emotions sharing). Singing primates, in general, may thus represent a convenient animal model to unravel the origins and mechanisms shaping the evolution of speech and musicality [13,14]. Gibbons, and lar gibbons specifically, are ordinarily found in *ex-situ* contexts, making controlled collection of acoustic data possible. Similarities exist between gibbon songs and human music, for instance the collective context of emission of the song [15] and the association of ritualized locomotor displays to the song [16,17]. Because of this, gibbons may be good models for unravelling the biological origin of musicality in our *taxon* [16].

We searched for isochronous patterns in lar gibbons' songs, specifically in female and male contributions to duet (figure 1*c*) and male solos (figure 1*b*). We hypothesized that gibbons may flexibly deploy and modulate rhythmic isochrony based on social context [4,12,18]. We extracted the inter-onset-intervals (t_k_), namely the duration of the interval separating the onset of a song element from the next one. Then, we calculated the ratios between two adjacent intervals (*r_k_* = *t_k_*/(*t_k_* + *t_k_*_+1_)), analysing their distribution to detect the existence of categorical rhythms (density peaks on specific, small-integer ratios; e.g. a 1 : 1 peak represents isochrony) [5,9]. We then probed the biological substrates of isochrony in this species, testing the effect of biomechanical constraints, in terms of call rate, in explaining rhythmic patterns of gibbons’ duet: calling fast is energetically expensive and a potential signal of signaller's quality [19]. Because duetting seems a coordinated display [18], we also investigated the role of interaction in shaping the songs' rhythmic structure. To do so, we first assessed the causality of one individual's phonation on the one of the co-singer. Then, as synchrony may underpin coordinated displays [4,14], we quantified the overlapped phonation between duetting females and males, predicting that shared advertisement purposes enhance co-singer's synchrony. Finally, we tested for a relationship between isochrony and synchrony in the two sexes. We predicted that isochrony and synchrony may be strictly linked, similarly to humans. We also expected that the sexual dimorphism in form and function of gibbons' song may translate into a sex-specific trade-off between isochrony and synchrony.

## 2. Material and methods

### (a) Animals and recordings

Six habituated gibbon familiar groups were followed, with a total of 12 individuals and 215 songs, and specifically 157 female contributions to duet, 157 male contributions to duet and 58 male solos. Four groups were inhabiting the forests of Huai Kha Khaeng Wildlife Sanctuary (Thailand), and two other *ex situ* groups were living at the Cappeller faunistic park (Corvigliano, Italy) and the Falconara zoo park (Ancona, Italy). The contribution of each group and gibbon to the final dataset is provided in the electronic supplementary material, table S1.

We recorded the animals between 6:00 am and 12:00 am using a Sennheiser ME67 microphone connected to a solid-state digital audio recorder Tascam DR-100MKII (44.1 KHz sampling rate). All vocalizations were recorded at 5–50 m distance from the animals, aiming the microphone toward the individual vocalizing to maximize recording quality.

### (b) Acoustic analyses

We edited the songs using Praat 6.0.14 and saved them as WAV audio files [20]. Field notes and video recordings allowed us to recognize and separate individual contributions to each song; each annotated contribution was subsequently saved as a single Praat *TextGrid*, an object featuring onset and offset of each note. A computing cluster (OCCAM [21]) processed all 196 768 vocal units via a custom Praat script and exported all onsets of song units from separate TextGrids into one .csv datasheet. We calculated the temporal interval between an onset and the next one, which defines an inter-onset interval (*t_k_*). We focused on all *t_k_* ≤ 5 sec, as this value is typically hypothesized as upper limit for meter perception and performance in humans and there is no quantitative evidence about an upper threshold on other apes [22]. We calculated the tempo frequency as the inverse of peak values of *t_k_* per song type (Hz). The ratio (*r_k_*) was then calculated between a *t_k_* and the next one, *t_k_*_+1_, as *t_k_*/(*t_k_* + *t_k_*_+1_).

### (c) Statistical analyses

#### (i) Testing isochrony per song type

To test the significance of the peaks of the *r_k_* density distribution falling in the vicinity of isochrony (corresponding to *r_k_* = 0.5) we followed the methodology in Roeske and colleagues [5] and De Gregorio *et al.* [9]. On-isochrony ratio ranges were centred on a 1 : 1 ratio (i.e. *r_k_* = 0.500), while the off-isochrony ones correspond to the peripheral range at the left and right sides of the on-isochrony range. Specifically, we took on/off-isochrony boundaries at 0.400, 0.440, 0.555, 0.600 (*r_k_* values) and counted, per individual contribution and per song type (female contribution to duet, male contribution to duet, male solo), the number of *r_k_* instances falling into the off-isochrony versus on-isochrony sectors of the curve. We calculated an *isochrony rate,* as the ratio between on- and off-isochrony observations per contribution.

Using a generalized linear mixed model (GLMM; R package *glmmTMB* [23]), we tested whether *r_k_* observation counts differed by song type in interaction with the type of interval (on- or off-isochrony interval). The response variable was the observations count (*r_k_*), which followed a Poisson distribution, and the individual contribution code was used as a random factor (electronic supplementary material, table S3). To test the significance of the full model, we built a null model comprising only the random factors and compared the full and the null with a likelihood ratio test (Anova with ‘Chisq’ argument [24]). We obtained *p*-values for each predictor using the R *summary* function and performed pairwise comparisons for each level of the explanatory variables with the *emmeans* [25] package (*p*-values adjustment with Tukey method).

We then tested the effect of song types (fixed factor) on *isochrony rate* (response variable), which is the ratio between the number of on-isochrony and the number of off-isochrony observations per contribution, using a GLMM (*lme4* package [26]). The song code was entered as random factor. *Isochrony rate* was log-transformed and followed a normal distribution. Full versus null models were compared with a likelihood ratio test (Anova with ‘Chisq’ argument [24]). We obtained *p*-values for each predictor using the R *summary* function and performed pairwise comparisons for each level of the explanatory variables with *emmeans* [25] package (*p*-values adjustment with Tukey method).

#### (ii) Call rate calculation and assessment of its effect on isochrony

We calculated the *call rate* (number of onsets/10 s) and recalculated *isochrony rate* on contributions chunks of 10 s. A GLMM tested whether song type (predictor) affected *call rate* values (response variable), with individual contribution code as random factor (electronic supplementary material, table S4). The response variable, once transformed as 1/(*call rate* +1), fit a Gaussian distribution. We built (*lme4* [26] package) a full model and a null model comprising only the random factors. We compared them with a likelihood ratio test (Anova with ‘Chisq’ argument [24]). We obtained *p*-values for each predictor using the R *summary* function and performed pairwise comparisons for each level of the explanatory variables with *emmeans* [25] package (*p*-values adjustment with Tukey method).

We tested the effect of *call rate* in interaction with song type (fixed factors) on *isochrony rate* (response variable) with a GLMM (*lme4* [26] package), where the individual contribution code was a random factor (electronic supplementary material, table S5). The response variable, once log-transformed, followed a Gaussian distribution. We built and compared full and null models with a likelihood ratio test [24]. We obtained *p*-values for each predictor using the R *summary* function and performed pairwise comparisons and *p*-adjustment (Tukey method) with *emmeans* [25] and slope estimation with *lsmeans* [27] packages.

We wanted to test whether high versus low *call rate* affected isochrony. To test this hypothesis, we partitioned the *call rate* values between low and high *call rate,* depending on whether these were below or above the median of the specific song type. We counted how many *r_k_* observations (response variable) for each song type (predictor) fell on or off peaks (predictor). We then built a GLMM model testing whether *r_k_* counts could be explained not only by song type and on or off peaks—similarly to previous models—but also whether this purported relationship was modulated by an interaction with low versus high *call rate* (*glmmTMB* [23] package; see electronic supplementary material, table S6). We compared full and null models with a likelihood ratio test [24]. We obtained *p*-values for each predictor using the R summary function and performed pairwise comparisons and *p*-adjustment (Tukey method) with *emmeans* [25].

#### (iii) Assessing causality between male and female's contributions

We converted each individual contribution into binary series. We binned contributions into intervals of 10 ms. We mapped the interval to a new time series containing a 1 when phonated, 0 otherwise. We performed a Granger causality test (GrangerTest function in R − lag = 5, order = 500), which assesses whether a time series is efficient in predicting another, between the female and male binary time series of the same song, in both directions (F → M, M → F). We then counted the number of occurrences where *p* < 0.001, for F → M and M → F causality directions, to infer whether one of the two sexes affects more than the other the partner's timing of vocalizations. We limited the causality analysis to songs recorded in the wild to minimize the effect of potential external disturbances that often occur in captivity (e.g. other species' singing, visitors’ presence).

#### (iv) Is duets’ overlap different from chance?

For every contribution recorded in the wild, we split songs' TextGrids into chunks of 3 min and calculated the overlapped phonation, summing the TextGrids’ tiers with a custom Python [28] script. We then normalized it in two ways (electronic supplementary material, figure S7). First, we measured the rate (from 0, where no overlap is present, to 1, where all phonation is overlapped) of overlapped phonation on the total phonation of an individual contribution. Second, we also calculated the *normalized overlap*, by dividing the overlap duration in seconds by the total duration of phonation of the two co-singers (duration of phonation of the male + phonation of the female). We simulated random duets permuting each chunk against the others. For biological plausibility, the randomized permutation was carried out only between different sexes. Random duets were randomly paired songs either from real couples or from female-male non-coupled individuals. In simulations, we calculated the overlap and the *normalized overlap* as for real duets. We then tested whether the real overlap (REAL) was different from chance (SIMULATED permutations) with a GLMM (*lme4* [26] package; electronic supplementary material, table S8). The response variable, the *normalized overlap*, was normally distributed. We used the group code of the two singers as the random factor. We compared *full* and *null* model with a likelihood ratio test [24]. We obtained *p*-values for each predictor using the R *summary* function and performed pairwise comparisons and *p*-adjustment (Tukey method) with *emmeans* [25].

#### (v) Correlation between overlap and isochrony

We investigated the effect of overlap on the *isochrony rate* with a GLMM (electronic supplementary material, table S9). Both *isochrony rate* and *normalized overlap* were calculated on the whole individual contribution. *Isochrony rate* was the response variable, while *normalized overlap* and sex, in interaction, were fixed factors. We tested for the interaction between overlap and sex, expecting potentially dimorphic effects of those variables on isochrony. We used the song code as a random factor. The *isochrony rate* followed a beta distribution, so we used the *glmmTMB* package to build the *full* and *null* models and compared them with a likelihood ratio test [24]. We obtained *p*-values for each predictor using the R *summary* function and performed pairwise comparisons and *p*-adjustment (Tukey method) with *emmeans* [25]. We performed slope estimation using the *lsmeans* [27] package.

For every model, we assessed normality, homogeneity (via function provided by R. Mundry), and number of the residuals; the *performance* [29] package tested for collinearity among fixed factors. Effect plots were produced with *sjPlot* [30] package.

## 3. Results

### (a) Lar gibbons deploy isochrony in both duets and solos

**Figure 2.**
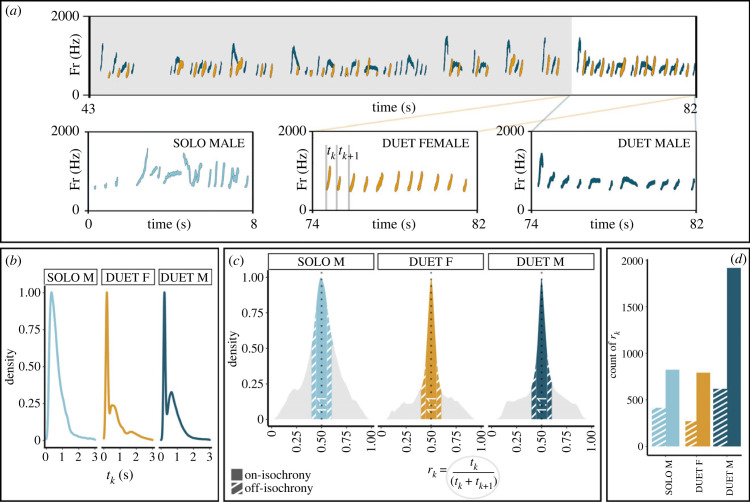
Rhythmic features of the lar gibbon's songs. (*a*) Zoomed-in portions of the spectrograms of both duet contributions and male solo. The fundamental frequency of individual contributions is highlighted in light-blue for the *male solo*, in dark-blue for the *male contribution to duet* and dark-yellow for the *female contribution to duet* (both detailed in specific portions indicated by the coloured lines). A representation of the onsets (grey lines) and the relative inter-onset intervals (*t_k_*) is reported on the spectrogram of the female contribution. (*b*) Probability density function representing the distributions of *t_k_* per song type. *t_k_* values are calculated on 12 adult individuals for a total of 372 individual contributions to songs. (*c*) Probability density function representing the distributions of rhythm ratios (*r_k_*) per song type, suggesting a difference between *male solos* and *male* and *female contributions to duet*s. Solid coloured sections of the curves indicate on-isochrony *r_k_* ranges, striped sections indicate off-isochrony *r_k_* ranges. (*d*) Histogram of the counts for *r_k_* values falling within on-isochrony versus off-isochrony ranges of the density function (depicted in panel *c*), per song type. For all song types, on-isochrony observations (solid bars) are significantly more numerous than off-isochrony ones (striped).

The distribution of raw inter-onset intervals (*t_k_* - figure 2*a*; electronic supplementary material, figure S2) values shows two peaks for female and male duet contributions (figure 2*b*), with maxima corresponding to *t_k_* values of 0.181 s (5.525 Hz) and 0.503 s (1.988 Hz) for the female duet, at 0.204 s (4.902 Hz) and 0.637 s (1.570 Hz) for the male duet. Male solos show only one peak at 0.322 s (3.105 Hz). Density plots of *r_k_* values (figure 2*c*) show a clear peak on the small integer-ratio of 1 : 1, namely isochrony. At least visually, there seems to be strong rhythmic regularity. We quantified this intuition by computing and comparing the count of r_k_ values falling into on/off-isochrony boundaries [9] (figure 1*d*). Through our model (*full* versus *null*: df = 5, Chisq = 24989.590, *p* < 0.001; electronic supplementary material, table S3), we found that the *r_k_* peaks around isochrony were statistically significant in the three song types (*female contribution to duet*, off- versus on-isochrony *r_k_* count: estimate = −1.168, *t*-value = −95.220, *p* < 0.001; *male contribution to duet*, off- versuson-isochrony *r_k_* count: estimate = −1.067, *t*-value = −106.792, *p*-value < 0.001; *male solo* off- versuson-isochrony *r_k_* count: estimate = −0.580, *t*-value = −34.293, *p* < 0.001), meaning that the number of *r_k_* values falling into on-isochrony range of the curve were significantly higher than those falling in the off-isochrony range.

### (b) Call rate, isochrony and their relationship are context-dependent

**Figure 3.**
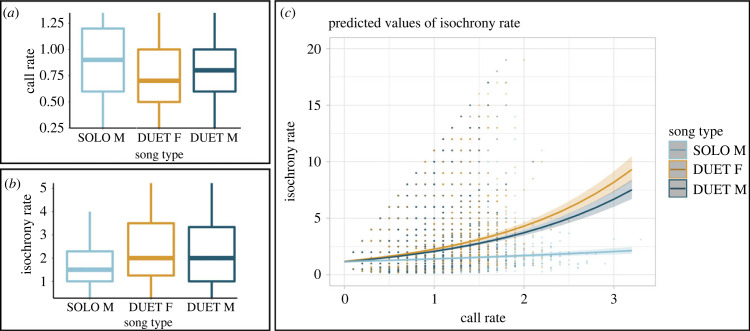
*call rate* and isochrony covariation in lar gibbon's song. (*a*) Boxplots representing the *call rate* per song type, calculated on chunks of 10 s for every individual contribution. The solo of the male shows significantly higher values of *call rate* than the male and female duets. Outliers corresponding to the 10% of higher and lower values of the variable are excluded from the plot. (*b*) Boxplots depicting the *isochrony rate* (on-isochrony counts/off-isochrony counts) per song type, calculated on chunks of 10 s for every individual contribution. The *isochrony rate* does not differ between male and female duet contributions, while the *isochrony rate* of the male singing solo is significantly lower than the one of the male and female in the duet. Outliers corresponding to the 10% of higher and lower values of the variable are excluded from the plot but included in the statistical computations. (*c*) Effect plot showing the predicted values derived from a GLMM looking at the effect of *call rate* on *isochrony rate*. The *female contribution to duet* and the *male contribution to duet* have a positive effect on *isochrony rate*, but both show a significantly steeper slope to the one of the *male solo*. Shaded areas indicate confidence intervals.

We obtained average *call rate* values (±s.d.) of 0.768 ± 0.360 Hz for the *female contribution to duet*, 0.774 ± 0.315 Hz for the *male contribution to duet* and 0.943 ± 0.468 Hz for the *male solo*. Our model testing the effect of song type on *call rate* values (*full* versus *null*: Chisq = 65.469, d.f. = 2, *p* < 0.001; electronic supplementary material, table S4) showed no rate difference between female and male contributions to the duet, but significantly higher values of *call rate* for the male solo (*post-hoc* comparisons; *female contribution to duet* versus *male contribution to duet*: estimate = 0.007, *z* ratio = 1.495, *p* = 0.293; *female contribution to duet* versus *male solo*: estimate = 0.046, *z* ratio = 8.056, *p* < 0.001; *male contribution to duet* versus *male solo*: estimate: 0.039, *z* ratio = 7.028, *p* < 0.001 – electronic supplementary material, figure S2 and table S4; *call rate* values showed in figure 3*a*). Our model (*full* versus *null*; Chisq = 1569.052, d.f. = 5, *p* < 0.001 – electronic supplementary material, table S5) testing the effect of *call rate* in interaction with the song type on the *isochrony rate* (figure 3*b*) showed no differences between female and male contributions in a duet (*post-hoc* comparisons; *f*e*male contribution to duet* versus *male contribution to duet*: estimate: 0.075, *z* ratio = 2.363, *p*-value = 0.048; fe*male contribution to duet* versus *male solo*: estimate = 0.383, *z* ratio = 9.442, *p*-value < 0.001 – electronic supplementary material, table S5). Conversely, we found significantly lower isochrony in the *male solo* compared to both the female and male duets (*post-hoc* comparisons; *male contribution to duet* versus *male solo*: estimate = 0.309, *z* ratio = 7.742, *p*-value < 0.001 – electronic supplementary material, figure S2 and table S5). Isochronous regularity differs between sexes in a duet—females are more isochronous—and either sex has more isochrony in duets than males have in solos. Male solos have higher call rates but lower isochrony. These two results showed a context-dependent level of isochrony in the male, with the male singing more regularly when duetting than when singing alone; they also suggest that higher levels of isochrony *cannot* exclusively derive from higher call rates. Our model shows that call rate has a significant positive effect on isochrony; however, song type modulates this effect (i.e. the solo shows a significantly less steep slope, and thus significantly weaker effect, than the duet contributions of male and female—figure 3*c*, electronic supplementary material, table S4). To summarize, male solos show higher call rates, lower isochrony and weaker correlation between these two variables than the other song types.

We tested whether high versus low levels of *call rate* modulated isochrony. All the isochronous peaks were still significant at both high and low values of *call rate* (electronic supplementary material, table S5), confirming that *call rate* alone cannot explain isochrony levels.

### (c) Male and female influence each other's phonation onsets

**Figure 4.**
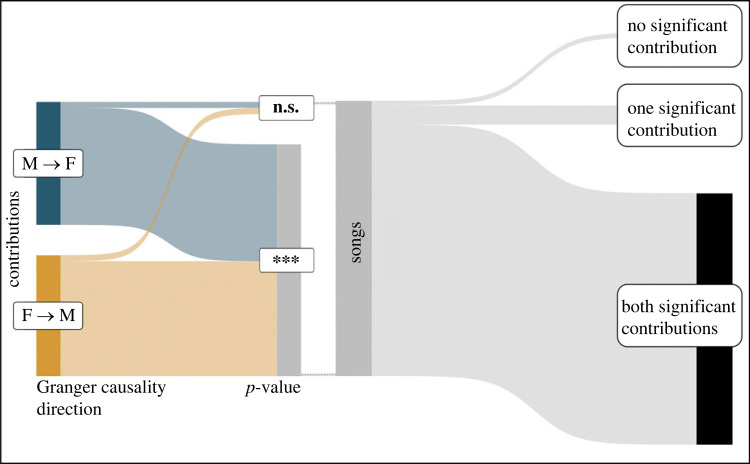
Granger causality in lar gibbon's duet—aggregated Sankey plots showing: on the left, the *proportion of contributions* having a significant causality (***p < 0.001) on the onsets of the co-singer, for both directions (male on female and female on male) and non-significant ones (n.s.); on the right, the *proportion of songs* showing a reciprocal causality (both male on female and female on male), having significant causality in only one-direction (male on female or female on male) or no significant causality at all.

Since *call rate* was not sufficient to explain context-dependent isochrony levels, we investigated the presence of rhythmic interaction, by testing whether an individual's vocal rhythm affects its partner's. For this we used Granger Causality, a test probing whether future vocal onsets of one individual can be better predicted by considering past onsets of the co-singer (as opposed to past vocal onsets of the first individual). We found that 95% of the individual contributions to duets affect (i.e. Granger-caused at *p* < 0.001– figure 4) the partner's phonation. Moreover, this effect emerged bidirectionally, both for the female on male's contribution (F → M in figure 4) and the male on female's contribution (M → F in figure 4). At the song level, 91.39% of the songs showed a reciprocal highly significant causality (*p* < 0.001) between male and female contributions, 6.89% showed at least one significant contribution, 1.72% showed no significant contribution. This provided compelling evidence that the two sexes influence each other's onsets during the duet.

### (d) Synchrony: duet overlap is higher than chance

**Figure 5.**
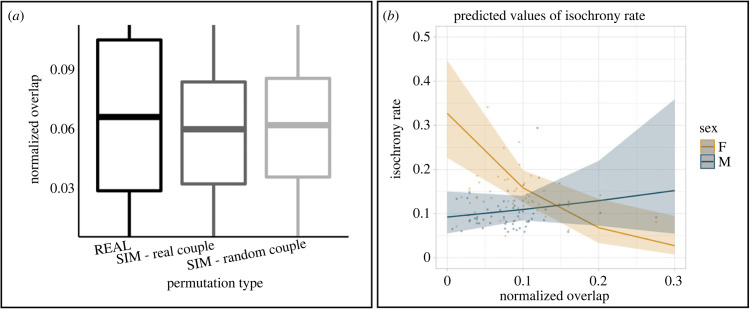
Synchrony and isochrony covariation in lar gibbon's song. (*a*) Boxplots showing the amount of *normalized overlap* (sum of all durations of overlapping female-male phonation / sum of all durations of female-male phonation), measuring the degree of synchrony. *Normalized overlap* is a rate going from 0 (no overlap between the co-singers) to 0.5 (co-singers perfectly overlapped). The amount of overlap is calculated for the real duets (REAL) and simulated ones (SIM - two types of simulations: simulated duets of real couples and simulated duets with random couples composed of opposite-sex individuals from the sample). Real duets show significantly higher overlap than simulated ones, displaying that the animals are statistically more synchronous than expected by chance. (*b*) Effect plot showing the predicted values taken from the GLMM looking at the effect of *normalized overlap* on *isochrony rate*. The two regression lines show the sex-specific trends that link the two variables. Shaded areas indicate confidence intervals.

Since we found a potential for rhythmic interaction, we then quantified overlap in the duets through two different metrics. The first metric, normalized on the individual total phonation, showed that females presented a rate (from 0 to 1) of overlap (mean ± s.d.) of 0.177 ± 0.145 while males of 0.158 ± 0.131. The second metric, the *normalized overlap* (normalized on the sum of phonations of both co-singers – a rate from 0 to 0.5), in actual duets (REAL; mean ± s.d. = 0.079 ± 0.037) assessed the degree of synchrony between actual singing partners (electronic supplementary material, table S8). To have baselines and quantify randomness, the *normalized overlap* was also calculated on the randomly permuted opposite-sex individuals' contributions, for both existing couples (SIMULATED: real couple; mean ± s.d. = 0.060 ± 0.037; electronic supplementary material, table S8) and randomly paired individuals (SIMULATED: random couple; mean ± s.d. = 0.062 ± 0.035; electronic supplementary material, table S8). When testing the difference in overlap (response variable) between real duets and randomly simulated ones (figure 5*a*; GLMM, *full* versus *null*; Chisq = 28.711, *p* < 0.001; electronic supplementary material, table S9), we found that real duets showed significantly higher overlap than both types of simulated ones (SIMULATED random couple – REAL estimate = −0.018; *z* value = −2.416; *p* = 0.037; SIMULATED real couple – REAL: estimate = −0.016; *z* value = −5.347; *p* < 0.001). The two types of simulated duets showed no difference in their rate of *normalized overlap*. These results indicate that the overlap, and thus synchrony, heard in actual duets is not perfect but significantly higher than chance.

### (e) The effect of synchrony on isochrony is sex-specific

Our model looking at the effect of synchrony (*normalized overlap*) and sex on isochrony (*isochrony rate*—*full* versus *null*; Chisq = 30.526, d.f. = 3, *p* < 0.001; figure 5*b*; electronic supplementary material, table S10) showed a significant effect of synchrony on the *isochrony rate* (estimate = −9.484, *t*-value =−2.416, *p* = 0.001; electronic supplementary material, table S10). In particular, we found a significant interaction between overlap and sex (estimate = 11.383, *t*-value = −5.347, *p* < 0.001; electronic supplementary material, table S10). There was a significant negative effect for females (confidence interval: lower cl. = −15.26; upper cl. = −3.71; electronic supplementary material, table S10)—which means higher isochrony corresponding to lower synchrony—and a non-significant positive trend for males (confidence interval: lower cl. = −3.37; upper cl. = 7.17; electronic supplementary material, table S10 figure S5b): the effect of synchrony on isochrony is overall significant but sex-specific.

## 4. Discussion

We provide compelling evidence for isochrony and synchrony in the song of an ape and elucidate the existing link between the two. Our results show how lar gibbon's songs are organized into highly isochronous, though plastic, rhythmic patterns; this isochrony is only partially explained by physiological constraints linked to call rate. Male inter-onset intervals become less variable and more isochronous from solo to duet, suggesting that the interaction between co-singers may shape the song's rhythmic structure. Furthermore, the timing of phonation of each individual in a duet is predicted by the timing of the co-singer's vocalizations, suggesting a potential rhythmic interaction. The exact coincidence of events in time can be quantified as the overlap between two individuals' vocalizations, namely their synchrony, which we showed is higher than chance across singers when compared with artificial dyads. Finally, we searched and found a link between synchrony and isochrony, specifically a significant statistical interaction indicating different trends across sexes. Females show a significant negative relationship between rhythm regularity and overlap, suggesting a potential trade-off between individual and group rhythms.

### (a) Isochrony and its role in coordinated displays

The production of isochronous rhythm in spontaneous animal vocalizations is rare and poorly studied [2,31]. Isochrony may have important biological functions: for instance, rock hyraxes producing more isochronous rhythmic patterns have higher fitness, in terms of reproductive success [8]. Here we provide evidence for above-chance occurrence of isochrony in an ape species. This occurs in both male and female songs. *t_k_* peak values in lar gibbons are dimorphic and bimodal (similarly to another primate, *Indri indri* [9]), and differ between song types. Gibbons’ isochrony transcends absolute temporal intervals: raw *t_k_* values may vary but nonetheless lead to the same isochronous 1 : 1 ratio across sexes and song types. That is to say, as in human music, isochrony emerges even when tempo or note duration vary [11]. At the same time, *t_k_* values are not randomly distributed, but mainly clustered around short intervals that produce the isochronous patterns: gibbon songs show two dominant tempi (1/*t_k_* peak values) in the duet contributions which are tentatively close to two human behaviours: music and speech. The slower tempo—at 1.988 Hz for the female and 1.570 Hz for the male—is closer to Western music, where 2 Hz is the preferred tempo [32] and human locomotion [33]. The faster tempo we found—at 5.525 Hz for the female and 4.902 Hz for the male—falls in the range of speech, which relies on the association of vocal output and facial posture in the 3–7 Hz range [34]. Similarly, macaques' ‘lip smacks’ [35] and orangutans' ‘clicks/faux-speech’ [36] have tempi close to speech. Speech-like rhythm in nonhuman primate vocalizations, often associated with facial expressions, may underpin—according to some—an ancestral original audiovisual rhythmic feature, still detectable in some branches of the primate phylogeny [35,36]. Gibbon tempi are numerically bounded by those of human music and speech. Beyond tempo, music and speech differ in rhythm regularity: music—like gibbon song—has it, while speech does not [37]. Human music and speech may have branched, says one hypothesis, from an ancestral ‘musical proto-language’ [38,39]; our finding of human-compatible song tempi in another ape provides indirect support to this hypothesis. Looking ahead, the sound-facial expressions link in gibbons is an open topic for future research, and so is the similarity between their songs' tempo and human music or speech.

The use of isochrony to engage in group coordination, as for choruses and dance, was deemed a uniquely human trait [4]. Some features of gibbons’ song are considered inherited and developmentally fixed [16,40]. Nonetheless, lar gibbons can instantly and flexibly adjust their vocal contribution; they can start and stop singing depending on subtle temporal and spectral variations in the co-singer utterances [18]. This may involve a duet coordination mechanism that potentially requires mutual learning and fine-tuned adaptation. Supporting this hypothesis, established pairs show higher organization and coordination when compared to new ones [41]. Our data also support this learning and adaptation hypothesis. We found a reciprocal causal link between the timing of the vocal emission of duetting individuals: the onset of one individual determines the onset of the co-singer. Moreover, males show higher isochrony in duets than in solos. We thus show that isochrony is deployed differently depending on social context, potentially fulfilling a need for tuning rhythmic structure via tempo regularity. Speculatively, if reciprocal rhythmic adaptation were a flexible and expensive learning process, it might represent an explicit energy commitment; this rhythm-advertised commitment may enhance the strength of the pair-bond and lower the risk of partner desertion [42].

Human experiments show that both music and speech display temporal regularity when people mutually share a coordination purpose [12]: regular rhythm may support cooperative interaction. Our results showing enhanced isochrony in gibbons duetting might imply that they share with us some signalling aims, such as cooperatively communicating and then being subjected to similar selective pressures. If so, they may also rely on homolog or analogue neural mechanisms that allow rhythm and coordination in humans.

Finally, physiological constraints in vocal emission may also partly explain our results. Solo songs are, on average, longer [15] and show a higher call rate than duets. It is likely that a longer vocal display and a high call rate demand higher energy investment and depend on breathing constraints [19]: vocal fatigue may determine less regular rhythmic patterns in prolonged bouts [8,43]. At the same time, a costly display like the song is an honest signal of the emitter quality [44], making solo songs highly subjected to sexual selection [45]. The flexibility of rhythmic structure may thus not only serve vocal interaction between co-singers, but also be the outcome of a trade-off between quality signalling and physiological constraints.

### (b) Overlap versus turn-taking: parallels with human music and speech

Across species, synchronous coordination stems from two primary purposes: either minimization of the overlap in favour of turn-taking or maximization of the overlap toward signal amplification [3,4]. We found a higher than chance rate of overlap in the songs. This finding dovetails with studies suggesting that overlap in the songs may mediate mechanisms such as mate attraction, anti-predatory purposes (e.g. gibbons [43], anurans [45]) and signalling the cohesion of an alliance in mammals (e.g. lemurs [46], dolphins [47]). In bottlenose dolphins, cooperative context enhances motor and vocal synchrony: a shared function may enhance synchrony and coordination [47]. Similarly, male and female gibbons jointly advertise their presence in the forest, and signal amplification through synchrony enhances transmission over long distances [14,48] which Merker *et al.* [4] hypothesized crucial in our ancestors' long-distance calls. Long-range signal transmission is needed to localize conspecifics and reduce the costs of territorial conflicts [49,50]. The adaptiveness of group signalling more than individual broadcasting, and the preponderance of synchrony rather than turn-taking, may be the result of a monogamous mating system [51]. The avoidance of overlap seems to be preeminent in morphologically dimorphic species subjected to higher levels of sexual selection [52]. Our results showing a higher than chance level of overlap in a monogamous, non-morphologically dimorphic species corroborate the idea that species subjected to weaker sexual selection [53] benefit from synchrony more than from turn-taking.

Human speech and music are notably different in the amount of overlap versus turn-taking occurring during interactions [54]. Both speech and music rely on rhythm. In conversations, speakers tend to minimize silence and avoid overlap: turn-taking is enhanced through spectro-temporal clues like prosody. Cross-cultural work showed that overlap avoidance and silence minimization, quantified via response latency in conversation, are the norm across languages [55] Conversely, ensemble music often shows high levels of overlap: in many musical cultures (near-)synchrony is enhanced. Both mechanisms involve flexibility, adjustment and anticipation that allow the two domains to serve their different, specific adaptive functions. Our gibbons exhibit an average overlap rate of 16–18%, with higher than chance level of synchrony. It is difficult to compare our overlap rates (obtained by diving duration overlap by duration of phonation of the singer) to those of human conversations (often expressed as duration of silent gaps in seconds) or music. However, qualitatively, we would argue that gibbon overlap rates are well above what observed in human spoken conversations, and below those characteristics of several musical genres [55].

### (c) Sex-specific relationship between isochrony and synchrony

Our results highlighted a complex relationship linking synchrony and isochrony. We found an overall negative effect of synchrony on isochrony on the whole duet, and dimorphic effects of synchrony on isochrony. Synchrony did not enhance isochrony in males, but significantly decreased isochrony in females. This supports the idea that songs serve multiple functions that vary depending on dimorphic selection pressures acting on the duet contributions [13,56–58]. We propose that, for both sexes, isochrony may function to increase the redundancy of the signal during communication [2] and to mediate the overlap between the co-singers aiming at signal summation [4]. A trade-off mechanism may explain how females’ isochrony decreases with higher synchronization: besides the interest in advertising the couple's mated status to neighbouring groups, females may broadcast their quality to higher quality males and female competitors. Previous work suggests that females' great calls are both an index of their physical condition [59] and the least overlapped part of the song [18]. Hence, we propose that female advertisement can be achieved by minimizing synchrony, hence changing the rhythmic structure of a bout, thus making it less predictable for their mate. In other words, the female would be the most rhythmically flexible [60] of the two in a race to the arms with its partner [61].

Some hypothesized human music originated via an ancestral form of loud call, shared with primates closely related to us [4,16]. Numerous extant ape species show vocal displays, e.g. loud calls and songs, that share features with human music and may also derive from a proto-musical loud call of our last common primate ancestor. Such features include loudness, for long-distance communication, tonal notes organized into higher-level structures, inked locomotor displays coordinated to vocal utterances and an inherited component [16]. After the divergence of the human branch, our species coordinated vocal displays specialized and acquired unique characteristics [16,62,63]: more flexibility in song structure improvisation and new conventions, a crucial role of learning shaping all those processes, the presence of a stable beat structuring rhythmic music structure. Our results offer indirect support to this hypothesis.

Isochrony is a ‘statistical universal’ feature of human music, meaning it is found more often than not across diverse musical cultures [11,64]. Some argue it may be rare in other species, or even a human prerogative [62]. Others maintain that isochrony's function for group coordination may be the one feature exclusive to our species [4]. Indeed, whether endogenous isochrony can be driven by exogenous (e.g. environmental, social) factors, in other species, is unknown [2], even in the only non-human mammals (*Indri indri*) shown to date to have rhythmic categories [9]. Our results not only support the idea that a primate closely related to us can spontaneously produce isochronous rhythms, but also that this rhythmic pattern can be shaped by exogenous *stimuli* linked to interaction. Endogenous and exogenous mechanisms coexist and are not mutually exclusive in determining rhythm [65]; consequently, rhythm should be investigated in terms of both underlying neural circuitry [66] and species-specific social and communicative adaptations [9]. From a comparative perspective, rhythmic capacities result from a mosaic of anatomic and functional changes throughout evolution. This perspective is in line with the *gradual audiomotor evolution* hypothesis, suggesting a coupling between the auditory and motor system in non-vocal-learners primate species [67]; the closer a primate is to humans, the more developed their rhythmic skills should be in terms of coordination and entrainment skills. Rhythmic capacities in gibbons support for the *gradual audiomotor evolution hypothesis*: our similar neural substrate, and potentially convergent pressures for singing, may grant similar rhythmic capacities. The rhythmic features we found are only a first step towards understanding potential rhythmic entrainment in gibbons.

Our results all together support the idea that evolution, by convergence or shared ancestral traits, may have selected isochrony as an adaptive trait for collective, coordinated vocal displays. Still, the role of isochrony in mediating synchronization in animal species remains widely unexplored. Now that individual building blocks of animal rhythms are increasingly studied, one can start probing their interaction. We suggest that elucidating the link between synchrony and isochrony may represent a substantial step in reconstructing musicality evolution and its meaning for our and other species.

## Data Availability

Timing data used for this study are available from the Dryad Digital Repository: https://doi.org/10.5061/dryad.wpzgmsbrm [68]. Code and custom-written scripts are available from the corresponding author, T.R., upon request. Additional tables and plots are provided in electronic supplementary material [69].
